# 1998 Dengue Hemorrhagic Fever Epidemic in Taiwan

**DOI:** 10.3201/eid1003.020518

**Published:** 2004-03

**Authors:** Day-Yu Chao, Ting-Hsiang Lin, Kao-Pin Hwang, Jyh-Hsiung Huang, Ching-Chuan Liu, Chwan-Chuen King

**Affiliations:** * National Taiwan University, Taipei, Taiwan; † Department of Health, Taipei, Taiwan; ‡ Kaohsiung Medical University, Taiwan; § National Cheng Kung University, Tainan, Taiwan

**Keywords:** dengue fever, dengue hemorrhagic fever, dengue shock syndrome

**To the Editor**: The rapid spreading of dengue viruses has led to increasing incidence rates of dengue fever (DF), dengue hemorrhagic fever (DHF), and dengue shock syndrome (DSS) worldwide in the past 20 years. The global pandemic of DF and DHF in 1998 was associated with the largest DF epidemics many tropical or subtropical countries had ever experienced (*1*,*2*). Here we report the unique epidemiologic characteristics of DF and DHF caused by dengue virus type 3 (DEN-3) in Taiwan, where dengue is not endemic.

The recent epidemics of dengue in Taiwan started when dengue virus type 2 (DEN-2) was first introduced into the southern off-islet of Hsiao-Liu-Chiu in 1981 after an absence of 38 years since World War II. Tainan City in southern Taiwan had not had a dengue epidemic since l942–1943 until three dengue outbreaks occurred there in the last decade. The first outbreak of DEN-1 in 1994 and the second of DEN-2 in 1997 involved few confirmed cases. The third epidemic of dengue, which was attributed to DEN-3, began in October 1998 and continued into January 1999.

From August 1, 1998, to January 31, 1999, physicians in all the hospitals and clinics in Tainan City were required to report any suspected dengue cases who met the criteria of fever (>38^o^C) and two or more of the following symptoms and signs: headache, retroorbital pain, myalgia, arthralgia, rash, and hemorrhagic manifestations. Patients who met the criteria were invited to participate in the study; informed consent was given by the patients, and plasma or serum samples were collected for laboratory confirmation. When a physician reported a suspected dengue case, a minimum of 100 blood samples would be collected from the patient’s neighbors by the Tainan City Health Bureau staff. The blood specimens were transported to the laboratory at the National Institute of Preventive Medicine for confirmation. A confirmed dengue case was required to be positive by either reverse transcription–polymerase reaction (*3*), or demonstrate seroconversion by dengue-specific immunoglobulin (Ig) M and seronegativity for Japanese encephalitis virus (JEV)-specific IgM by IgM-enzyme-linked immunosorbent assay (IgM-ELISA) (*4*). Dengue viruses were isolated in C6/36 cell cultures and identified to serotype with monoclonal antibodies (*5*). The clinical diagnosis of DHF was based on the World Health Organization’s (WHO) criteria that were revised in 1997. Those confirmed dengue cases were classified as primary, secondary, or indeterminate infections, depending on the ratio of dengue-specific IgM/IgG as measured by the capture IgM and IgG ELISA (*6*).

Of 225 case-patients with suspected dengue, 142 patients, of which 74 (52.5%) were female and 68 (48.2%) were male (62.7%), had their cases confirmed by laboratory diagnosis during the study period. Their ages ranged from 7 to 79 years (mean: 39 years). Of the 23 DHF case-patients meeting the WHO’s case definition, 17 were male. The ages of the DHF case-patients ranged from 13 to 73 years, with a mean age of 42 years.

The epidemic began in October and peaked in November. In the Central District, where the earliest and majority of DHF cases occurred (52.2%), the DHF/DF ratio increased with time, from 11% during the first interval, to 20% and 30% during the second and third intervals, respectively. Although this increase was not statistically significant by Fisher’s exact test because of the small sample size, similar results were reported in Cuba’s DHF epidemic in both 1981 and 1997 (*7*). Many RNA viruses, such as influenza, increase in virulence through transmission; possible virulence mechanisms of dengue viruses are now under investigation. In other words, the duration of epidemic has to be as short as possible to avoid the emergence of DHF cases in that region.

In our study, 88 dengue cases (62%) were classified as primary infections, 32 (22.5%) as secondary infections, and 22 (15.5%) as undetermined because of the lack of paired acute- and convalescent-phase samples. DHF cases showed no significant association with secondary infections (odds ratio = 1.92 [95% CI 0.64 to 5.76], p = 0.19). Because the last documented transmission of dengue viruses in Taiwan occurred during the island-wide dengue epidemic in 1943, both DF and DHF cases were stratified into two birth cohort groups. Of DF case-patients born after 1943, 94% (83/88) had primary infection, and 11 (92%) of 12 DHF case-patients had primary infection. By contrast, 84% (27/32) of all the dengue case-patients born before and during 1943 had secondary infection, and 7 (78%) of 9 DHF case-patients had secondary infections. After the cohorts were stratified by age, DHF cases were not associated with secondary infection (Mantel-Haenszel) weighted odds ratio: 0.84 [95% CI: 0.11 to 5.62]) (p = 0.6). Therefore, age was not a confounding factor or effect modifier for DHF case-patients in Tainan's 1998 epidemic. DHF cases were mostly associated with primary infection in Tainan, where no large-scale epidemic of dengue had been reported from 1944 to 1997.

Our observation of DHF in adult case-patients was different from that in dengue hyperendemic Asian countries where 80% of DHF case-patients are children (*8*). Dengue virus type 3 was the only serotype isolated during the epidemic in Tainan in 1998. However, this situation was similar to that in Tonga in 1974, where a dengue virus was also newly introduced (*9*), and to recent epidemics in south and central America (*10*). More adults may have been affected because fewer dengue epidemics, and therefore fewer exposures to dengue viruses during childhood, had occurred; subsequently, the immune status in adults had changed. Presumably, previous observations of severe dengue in children in Southeast Asia were the result of immunity to infection in the older population, rather than a particular susceptibility to DHF among children. Our results were not influenced by the age structure of DF and DHF case-patients in cases of indeterminate infection or in cases of sub-clinical infection in children (0.4%, unpub. data). The dengue virus with epidemic potential replicated to higher viremia titer and was associated with disease severity without consideration of immune status (*11*). The investigation on molecular evolution of DEN-3 virus during the 1998 epidemic in Taiwan, currently in progress, will elucidate the possible role of virus variation in the pathogenesis of DHF.
